# Presence and Analysis of Plasmids in Human and Animal Associated *Arcobacter* Species

**DOI:** 10.1371/journal.pone.0085487

**Published:** 2014-01-20

**Authors:** Laid Douidah, Lieven De Zutter, Filip Van Nieuwerburgh, Dieter Deforce, Hanne Ingmer, Olivier Vandenberg, Anne-Marie Van den Abeele, Kurt Houf

**Affiliations:** 1 Department of Veterinary Public Health and Food Safety, Faculty of Veterinary Medicine, Ghent University, Merelbeke, Belgium; 2 Laboratory of Pharmaceutical Biotechnology, Faculty of Pharmaceutical Sciences, Ghent University, Ghent, Belgium; 3 Department of Veterinary Disease Biology, Faculty of Health and Medical Sciences, Copenhagen University, Frederiksberg, Denmark; 4 National Reference Centre for *Campylobacter*, Department of Microbiology, Iris-Lab, Brussels Public Hospital Network, Brussels, Belgium; 5 Laboratory of Microbiology, Sint-Lucas hospital, Ghent, Belgium; Niels Bohr Institute, Denmark

## Abstract

In this study, we report the screening of four *Arcobacter* species for the presence of small and large plasmids. Plasmids were present in 9.9% of the 273 examined strains. One *Arcobacter cryaerophilus* and four *Arcobacter butzleri* plasmids were selected for further sequencing. The size of three small plasmids isolated from *A. butzleri* and the one from *A. cryaerophilus* strains ranged between 4.8 and 5.1 kb, and the size of the large plasmid, isolated from *A. butzleri,* was 27.4 kbp. The G+C content of all plasmids ranged between 25.4% and 26.2%. A total of 95% of the large plasmid sequence represents coding information, which contrasts to the 20 to 30% for the small plasmids. Some of the open reading frames showed a high homology to putative conserved domains found in other related organisms, such as replication, mobilization and genes involved in type IV secretion system. The large plasmid carried 35 coding sequences, including seven genes in a contiguous region of 11.6 kbp that encodes an orthologous type IV secretion system found in the *Wolinella succinogenes* genome, *Helicobacter pylori* and *Campylobacter jejuni* plasmids, which makes this plasmid interesting for further exploration.

## Introduction

Arcobacters are small Gram-negative, aerobic to microaerobic bacteria belonging to the family *Campylobacteraceae* within epsilonproteobacteria [1]. To date, the genus comprises 15 species, has a widespread distribution in the environment, and a broad range of animal hosts. The species *Arcobacter butzleri*, *Arcobacter cryaerophilus* and *Arcobacter skirrowii* are classified as potential food and waterborne pathogens for both humans and animals [2]. *Arcobacter butzleri* and *A. cryaerophilus* are the species that are mostly associated with intestinal disease in humans [1], [3], [4]. The main symptoms of an infection are a watery, persistent diarrhea, nausea, and vomiting. In addition, *A. butzleri* and *A. cryaerophilus* have also been suggested to cause septicemia [5]–[9]. The epidemiology as well as virulence mechanisms of *Arcobacter* in human and animal disease are however not well established.

Plasmids are commonly present in diverse prokaryotes and play an important role in the genetic evolution and adaptation of bacteria. The acquisition of plasmids is a major factor in the ability of bacteria to exploit new environments and hosts [10]. This adaptation capacity can be attributed to the presence of genes coding for certain antibiotic, toxic heavy-metal, and radiation resistance, for the degradation of xenobiotic compounds, for virulence determinants or bacteriocin production, or for an increased mutation frequency [11]–[13]. Plasmids can also carry the genetic information for a type IV secretion system that has a role in gene transfer such as in the (Ti) plasmid in virulent *Agrobacterium tumefaciens*. This bacterium contains a large tumor-inducing plasmid that causes neoplastic transformation of the wounded tissue of a wide range of dicotyledonous plants [14].

Only few studies have reported the presence of plasmids in arcobacters so far [15]–[16]. In *Arcobacter butzleri* isolates from broiler carcasses and identified by the ApiCampy® system, plasmids with different sizes were detected [15]. In the study, no correlation was found between antimicrobial resistance and the presence of plasmids.

At present, little is known about the occurrence and function of plasmids in the human and animal associated *Arcobacter* species. Therefore, a total of 263 *A. butzleri*, *A. cryaerophilus*, *A. skirrowii* and *A. thereius* strains were examined for the presence of plasmids. Plasmids were extracted and sequence analysis was performed in order to assess their role in *Arcobacter* metabolism and pathogenicity.

## Materials and Methods

### *Arcobacter* Isolates from Humans, Animals and Food

To assess the presence of plasmids in a large collection of *Arcobacter* isolates: 10 *A. butzleri* and one *A. cryaerophilus* isolates from human patients were isolated between January 1995 to December 2002 at the National Reference Center for Enteric *Campylobacter*, Department of Microbiology, Saint-Peter University Hospital, Brussels, using the non-selective membrane filtration technique [3]. These isolates are classified as “historical strains” as they have been isolated more than a decade ago, and already used and published in several studies [3]. We are not aware of the ethical arrangements made at that time.

In addition, 13 *A. butzleri* and 11 *A. cryaerophilus* isolates from stool of adult and infant patients were obtained using the *Arcobacter* selective isolation method of Houf *et al.* [17] between October 2008 to December 2012 at the Saint-Lucas Hospital, Ghent [18]. For this, the advice of the institutional ethical committee has been asked and agreed on, and patient written consents are available. The identity of the patients was not revealed. Ninety-six *A. butzleri*, 82 *A. cryaerophilus,* 29* A. skirrowii*, and 21 *A. thereius* isolates were isolated from food and feces of food producing animals in the Department of Veterinary Public Health, Ghent University, Belgium, using *Arcobacter* selective isolation methods for food and feces [17]–[19]. Isolates from animals were taken from feces of animals with natural infection, and no experiments have been conducted. Four *A. butzleri*, four *A. cryaerophilus* and two *A. skirrowii* strains were recently isolated from horse and sheep feces [19].

### Identification and Typing

All isolates were subcultured onto blood agar plates and incubated for 48 h at 28°C under microaerobic conditions by evacuating 80% of the normal atmosphere and introducing a gas mixture of 8% CO_2_, 8% H_2_ and 84% N_2_ into the jar. Cell suspensions were prepared in 10 ml of sterile water with an optical density of about 0.074±0.002 (measured at 660 nm) which corresponded to a concentration of approximately 10^7^ cfu/ml. Template DNA was extracted from a 0.5 ml cell suspension of each isolate in phosphate buffered saline (PBS, Sigma-Aldrich, Irvine, Ayrshire, UK). Before extraction, all cell suspensions were centrifuged for 5 min at 17900 g (Eppendorf model 5417-R centrifuge, Hamburg, Germany country) to pellet the cells and the supernatants were discarded. The pellets were resuspended in 100 µl Tris-EDTA buffer and genomic DNA was extracted by the guanidiniumthiocyanate method described by Pitcher *et al*. [20]. Five µl of each DNA preparation was size-separated by electrophoresis in 1% gels to evaluate the integrity of the DNA extracted. The concentration of each DNA template was determined spectrophotometrically at *A*260 and adjusted to 50 ng µl^−1^. The DNA templates were stored at −20°C.

For identification at species level, an *Arcobacter* species-specific multiplex-PCR assay developed by Douidah *et al*. [21] was performed in a reaction mixture of 50 µl final volume composed of water (W4502, Sigma-Aldrich), 5 µl 10× PCR buffer (Invitrogen, Carlsbad, USA), 1.5 U Taq polymerase (Invitrogen) and a deoxynucleotide triphosphate mixture at a final concentration of 0.2 mM each (Invitrogen), 1.5 mmol of MgCl2 and 50 pmol of each primer ButR (5′-TCCTGATACAAGATAATTGTACG-3′), SkiR (5′-TCAGGATACCATTAAAGTTATTGATG-3′), TherR (5′-GCAACCTCTTTGGCTTACGAA-3′), CibR (5′-CGAACAGGATTCTCACCTGT-3′), ArcoF (5′-GCYAGAGGAAGAGAAATCAA-3′), GyrasF (5′-AGAACATCACTAAATGAGTTCTCT-3′) and GyrasR (5′-CCAACAATATTTCCAGTYTTTGGT-3′) [21]. The PCR assay involved 30 cycles of denaturation (94°C, 45 s), primer annealing (58°C, 45 s) and chain extension (72°C, 2 min). *Arcobacter* isolates that did not react in the multiplex-PCR were subjected to partial 16S rDNA sequencing.

To avoid the inclusion of identical strains, all isolates were further characterized below species level by a modified enterobacterial repetitive intergenic consensus (ERIC)-PCR [22]. Therefore one µl of DNA extract was added to 49 µl PCR volume. The ERIC motifs 1R 5′-ATGTAAGCTCCTGGGGATTCAC-3′ and 2 5′-AAGTAAGTGACTGGGG TGAGCG-3′ were used at concentrations of 25 pmol each. The PCR products were size separated by electrophoresis in 2% agarose gels in TBE buffer at 100V for 2 h. The banding patterns used to determine the genotypes comprised DNA fragments between 100 and 2072 bp. Computer based normalization and interpolation of the DNA profiles and numerical analysis using the Pearson product moment correlation coefficient, with 1% position tolerance, were performed using the GelCompar 4.2 software package (Applied Maths, Sint-Martens-Latem, Belgium). Dendrograms were constructed using the unweighted pair group linkage analysis method (UPGMA). For convenience, the correlation level was expressed as a percentage similarity. As shown in previous studies, DNA patterns that differed in one or more DNA-fragments were regarded as different genotypes [22], [23].

### Plasmid Detection and Extraction

Plasmids were extracted using the ZEPPY^Tm^ plasmid mini prep kit (Cat. No. D 4037, ZYMO RESEARCH, Irvine, USA) according to the manufacturer’s instructions. Ten µl of plasmid DNA extract was size separated by electrophoresis in a 1% agarose gel with 1X TBE for 120 min at 120 V, followed by staining in 1 µg/ml ethidium bromide. An UV transilluminator and photograph system (MICROdoc, Cleaver Scientific, Ltd) with an analyst computer program (Easy software, Kodak) was used for visualization.

### Restriction Enzyme Profiles

A restriction profiling of the extracted plasmids was first performed to select different plasmids for further sequence analysis. Therefore, the plasmid DNA was digested using the restriction enzymes *Kpn*I, *Hin*DIII, *Eco*RI, *Taq*I (Invitrogen). All digestions were performed in a reaction mixture of 20 µl, containing 10 µl of plasmid DNA extract, 20 units of endonuclease restriction enzyme, and 1x buffer. All mixtures were incubated for 5 hours at optimal enzyme temperature. All digested products were loaded and size-separated in 2% agarose gels in 1x Tris-borate-EDTA buffer at 120 V for 120 min, visualized as described above.

### Plasmid Extraction for Further Sequencing

Based on the enzyme restriction profiles, different plasmids were selected for further sequencing. Therefore, high-quality plasmid extraction was performed using plasmid midi Qiagen kit (Cat. No. 12143, Qiagen, Hilden, Germany) according to the manufacturer’s instructions. Plasmid DNA extraction was confirmed spectrophotometrically at A260 (Biophotometer, Eppendorf AG, Hamburg, Germany), assuring sufficient quantity and purity for sequencing.

### Plasmid Sequencing and Sequence Assembly

Roche GS-FLX titanium libraries were generated starting from 5 micrograms of purified plasmid DNA per sample. The DNA was fragmented by nebulization, followed by a double Solid Phase Reversible Immobilization (SPRI) bead capture size selection with Ampure beads (Agencourt Bioscience, Beverly Massachusetts, USA) to generate DNA fragments of 400–1,500 bp in length. The selected fragments were end-repaired and ligated to 454 multiplex identifier (MID) adapters to create a single stranded library which was used to perform an emulsion-based clonal amplification according to the Roche GS FLX titanium series emulsion PCR (emPCR) Method Manual – Lib L, version October 2009. The 4 resulting bead libraries from the smaller plasmids Ac1163, Ac1166, Ac637 and Ac1167 were pooled and sequenced on 1/8^th^ of a picotiter plate according to the Roche GS FLX titanium Sequencing Method Manual, version October 2009. The bead library from the larger plasmid Ac1119 was sequenced in a separate 1/8^th^ of a picotiter plate. The Roche GS *De novo* assembler version 2.6 was used to perform a *de novo* genome assembly. *De novo* assembly of circular genomes often results in contigs with overlapping ends. When this was the case, the overlapping part was manually trimmed. The Roche GS Reference Mapper was used to double-check this trimming. The results showed that all contigs were correctly trimmed and circular.

### Bioinformatics Analyses of Plasmids and Annotation

Following the construction of a single contig of each *Arcobacter* plasmid, the sequences were submitted for automatic gene annotation using the Rapid Annotation System Technology (RAST) server (http://rast.nmpdr.org) [24]. The annotation is based on subsystems, fully automated service for annotating bacterial and archaeal genomes. The putative coding sequences (CDSs) were identified using GLIMMER2 [25]. The RAST server also allows a comparative analysis using BLAST as tools to perform the analyses of similarity of the putative proteins in NCBI data. Mauve 2.3.1 was used to compare plasmid alignments of the four similar plasmids [26].

## Results and Discussion

Overall, plasmids were present in only 9.9% of the 273 examined *Arcobacter* strains. Ten percent of the *A. butzleri* strains isolated from poultry products (n = 80), and pig feces (n = 11) harbored plasmids, while no plasmids were detected in *A. butzleri* strains isolated from humans, cattle, sheep and horses (Table 1). The highest number of plasmids (20%) was detected in *A. cryaerophilus* strains isolated from pigs (n = 71). One of the four *A. cryaerophilus* strains from cattle also contained a plasmid. In *A. skirrowii*, plasmids were detected in 2 and 1 strains isolated from cattle and pig, respectively. No plasmids were detected in *A. thereius*. The enzymatic digestion patterns obtained by the enzymes *Kpn*I and *Eco*RI were not discriminative enough for all tested plasmids. In contrast, the enzymes *Hin*DIII and *Taq*I were more suitable for the analysis of *Arcobacter* plasmids (data not shown). In this study, plasmids with the same molecular size showed identical digestion patterns with all enzymes. Small plasmids up to 5 kbp were detectable in 26 strains. Only one large plasmid was present in an *A. butzleri* strain isolated from poultry products. The sequence length of the remainder plasmids were estimated by the use of digestion patterns of the plasmid DNA using different restriction enzymes and the gel electrophoresis profile of extracted DNA (Table 1). Multiple plasmids in a single strain were not detected.

**Table 1:** The occurrence of plasmids in *Arcobacter* strains from different matrices.

	Biological origin	N° of strains examined	Number of strains with plasmid	Plasmid size Kbp
*A. butzleri*	human	23	0	
	chicken	80	8	3, 4.8, 4.9(3x), 5.2(2x), 27.4
	pig	11	1	5
	cattle	5	0	
	sheep	2	0	
	horse	2	0	
*A. cryaerophilus*	human	12	0	
	chicken	7	0	
	pig	71	14	2, 4, 5(11x)
	cattle	4	1	5
	sheep	3	0	
	horse	1	0	
*A. skirrowii*	pig	13	1	5
	cattle	16	2	5
	sheep	2	0	
*A. thereius*	pig	21	0	

### Sequence Assembly

The sequence coverage for plasmid 6666666.8381 (*A. butzleri*, AC1119; GenBank accession number KF740630) was 1002x, 62642 of the 90684 generated sequences were assembled into one relevant contig of 27476bp. For plasmid 6666666.9998 (*A. cryaerophilus* strain R637, GenBank accession number KF740634), 1515 of the 1794 generated sequences were assembled into one relevant contig of 5104bp with the sequencing coverage of 85x. The coverage sequence for plasmid 6666666.8383 (*A. butzleri* strain AC1167; GenBank accession number KF740631) was 398x, 5429 of the 7291 generated sequences were assembled into one relevant contig of 4902bp. For plasmid 6666666.8384 (*A. butzleri* strain AC1166; GenBank accession number KF740632), 1242 of the 1313 generated sequences were assembled into one relevant contig of 4844 bp with The coverage sequencing of 69x. Finally, plasmid 6666666.8385 (*A. butzleri* strain AC1163; GenBank accession number KF740633), 2181 of the 2342 generated sequences were assembled into one relevant contig of 5153bp with the sequencing coverage of 135x. The start and the end of each sequenced plasmids showed significant overlap and represented the complete, circular plasmid.

The sequences of the five plasmids in the present study were compared with a cryptic plasmid (AP012049) detected in an *A. butzleri* strain [16], but showed to be totally different and shared no sequence homology.

### Small Plasmids

Based on the digestion patterns, five different plasmids were selected for further sequencing in order to investigate a maximum diversity and sequence content. For this, one *A. cryaerophilus* (R637) and four *A. butzleri* (AC 1119; 1163; 1166; 1167) plasmids were selected. The size and G+C content of the three small *A. butzleri* plasmids were 5.1 kbp (G+C = 25.8%), 4.8 kbp (G+C = 26.1%) and 4.9 kbp (G+C = 26.2%), isolated from strains AC1163, AC1166 and AC1167 respectively. The plasmid from *A. cryaerophilus* strain R637 was 5.1 kbp large and the G+C content was 25.4%. Sequence analysis of plasmids 6666666.8383 (AC1167; KF740631) and 6666666.9998 (R637; KF740634) using RAST server showed six ORFs and in plasmids 6666666.8384 (AC1166; KF740632) and 6666666.8385 (AC1163; KF740633), eight ORFs occurred (Table 2). In three small *A. butzleri* plasmids, a putative replication gene was found that was 54% similar to that in the P3386 plasmid of *Campylobacter coli* 338. In the *A. cryaerophilus* plasmid (R637), a putative replicase gene was detected that showed 59% similarity to the putative repB gene in *A. butzleri* and *Campylobacter hyointestinalis*. In the small plasmids AC1166 and AC1167, a putative diguanylate cyclase protein was characterized that showed 63% similarity to the diguanylate cyclase protein in *Hydrogenobaculum* species (GenBank accession number Y04AAS1), and 59% similarity with a conserved hypothetical protein in *Nitratiruptor* species (GenBank accession number SB155-2). The DNA sequence of a putative diguanylate cyclase was also detected in the other small plasmids AC1163 and R637 with a similarity of 99%. A putative mobilization gene was found in plasmids AC1166 and AC1163 in the coding sequences (CDS) fig|6666666.8384.peg.7 (AC1166) and fig|6666666.8385.peg.4 (AC1163) respectively. The protein shows 42% and 46% similarity to a putative mobilization protein located in *Campylobacter lari* and *Flavobacterium branchiophilum* respectively. Other putative genes were also detected but no theoretical function could be attributed to those putative ORFs (Table 2).

**Table 2:** Annotation of the four small plasmids using RAST server.

Strain	Feature ID	Start	Stop	Length (bp)	Function
*A. cryaerophilus* R637	fig|6666666.9998.peg.1	896	1972	1077	hypothetical protein
	fig|6666666. 9998.peg.2	2218	2054	165	hypothetical protein
	fig|6666666. 9998.peg.3	2428	2225	204	hypothetical protein
	fig|6666666. 9998.peg.4	3080	2445	636	hypothetical protein
	fig|6666666. 9998.peg.5	3571	3137	435	Initiator RepB protein family
	fig|6666666. 9998.peg.6	4390	3938	453	hypothetical protein
	fig|6666666. 9998.peg.7	4977	4579	399	hypothetical protein
*A. butzleri* AC1163	fig|6666666.8385.peg.1	2	205	204	hypothetical protein
	fig|6666666.8385.peg.2	212	376	165	hypothetical protein
	fig|6666666.8385.peg.3	1896	820	1077	hypothetical protein
	fig|6666666.8385.peg.4	2399	3289	891	mobilization protein
	fig|6666666.8385.peg.5	3505	3897	393	hypothetical protein
	fig|6666666.8385.peg.6	4217	5017	801	putative Rep
*A. butzleri* AC1166	fig|6666666.8384.peg.1	271	423	153	hypothetical protein
	fig|6666666.8384.peg.2	1051	497	555	diguanylate cyclase (GGDEF domain)
	fig|6666666.8384.peg.3	1287	1048	240	hypothetical protein
	fig|6666666.8384.peg.4	1573	1253	321	hypothetical protein
	fig|6666666.8384.peg.5	1717	1944	228	hypothetical protein
	fig|6666666.8384.peg.6	2075	2284	210	hypothetical protein
	fig|6666666.8384.peg.7	2305	3120	816	hypothetical protein
	fig|6666666.8384.peg.8	3131	3574	444	hypothetical protein
	fig|6666666.8384.peg.9	3934	4830	897	putative Rep
*A. butzleri* AC1167	fig|6666666.8383.peg.1	2	205	204	hypothetical protein
	fig|6666666.8383.peg.2	212	376	165	hypothetical protein
	fig|6666666.8383.peg.3	455	574	120	hypothetical protein
	fig|6666666.8383.peg.4	782	669	114	hypothetical protein
	fig|6666666.8383.peg.5	1224	763	462	diguanylate cyclase (GGDEF domain)
	fig|6666666.8383.peg.6	1444	1265	180	hypothetical protein
	fig|6666666.8383.peg.7	1748	1428	321	hypothetical protein
	fig|6666666.8383.peg.8	1892	2194	303	hypothetical protein
	fig|6666666.8383.peg.9	2244	2477	234	hypothetical protein
	fig|6666666.8383.peg.10	2688	3635	948	hypothetical protein
	fig|6666666.8383.peg.11	3999	4895	897	putative Rep

### Large Plasmid

**Figure 1:**
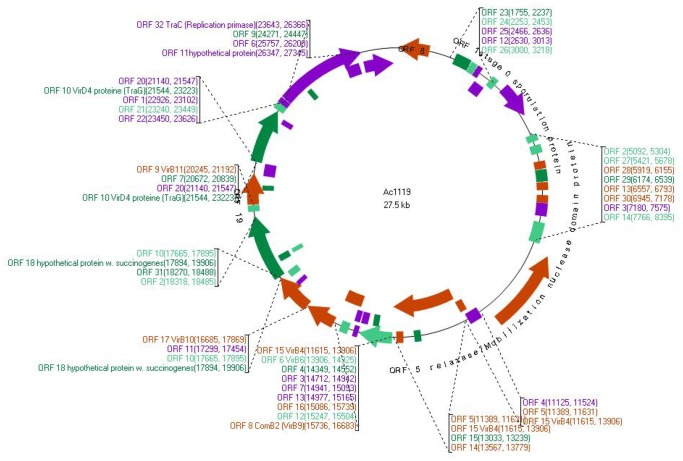
Physical map of the large *A. butzleri* plasmid (Ac1119). The hypothetical proteins and predicted ORFs are presented by colored boxes.

**Figure 2:**
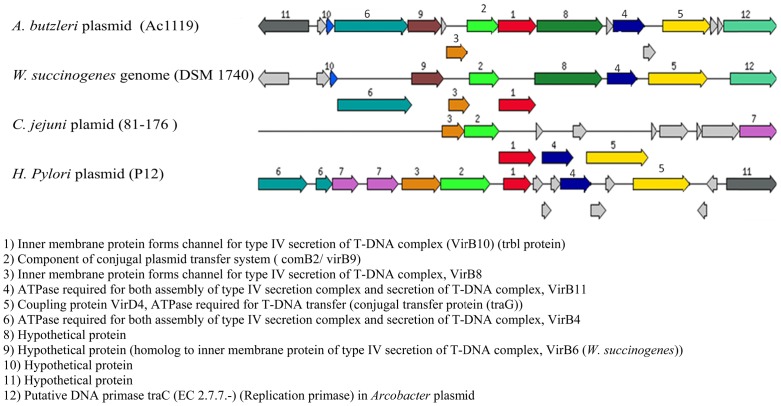
Type IV secretion system homology in the related organism.

A 27.5 kbp plasmid with a G+C content of 25.6% was detected in one *A. butzleri* strain isolated from broiler skin. A total of 35 ORFs were detected (Table 3, Figure 1). Eight of those encode putative proteins with extensive homology to proteins involved in a type IV secretion system in *Wolinella succinogenes* DSM 1740. Moreover, some of these proteins are also found in *Campylobacter jejuni* (pVir) and *Helicobacter pylori* p12 plasmids (Figure 2). The putative protein peg21 shows a similarity of 61% with the VirB4 gene detected in *W*. *succinogenes* (ATPase required for both assembly of type IV secretion complex and secretion of T-DNA complex). Peg26 is a putative protein involved in DNA or protein secretion in type IV secretion system and it shows similarity of 57% to the VirB10 gene in *W. succinogenes*. The protein Peg 29 shows 66% homology to VirB11 located in the same strain, an ATPase required for both assembly of type IV secretion and the translocation and secretion of T-DNA complex. The ORFs peg22, peg24 and peg25 are also putative proteins involved in the type IV secretion apparatus with homology to a plasmid conjugal transfer protein VirB6 in *Arcobacter nitrofigilis* DSM 7299 (66%), and in *W. succcinogenes* (49%), to VirB8 in *W. succinogenes* (63%), *H. pylori* and *C. jejuni* (56%) and to VirB9 in *W. succinogenes* (62%) and *H. pylori* (50%). Peg3 is a protein with homology to the conjugal transfer protein (TRAG) in *W. succinogenes* (65%), *H. pylori* (62%) and *C. jejuni* (61%). Peg34 is a protein similar to the TraC protein (coding for DNA replication primase), and is highly similar to the putative protein that was observed in *Yersinia pseudotuberculosis* (55%) and *Helicobacter pullorum* (MIT 98–5489) (46%).

**Table 3:** Annotation of the large plasmid AC1119, isolated from *A. butzleri* using the RAST server.

Feature ID	Start	Stop	Length (bp)	Function
fig|6666666.8381.peg.1	961	20	942	hypothetical protein
fig|6666666.8381.peg.2	1296	1183	114	hypothetical protein
fig|6666666.8381.peg.3	1653	2237	585	hypothetical protein
fig|6666666.8381.peg.4	2253	2453	201	hypothetical protein
fig|6666666.8381.peg.5	2466	2636	171	hypothetical protein
fig|6666666.8381.peg.6	2630	3013	384	hypothetical protein
fig|6666666.8381.peg.7	3000	3218	219	hypothetical protein
fig|6666666.8381.peg.8	3463	4479	1017	Chromosome (plasmid) partitioning protein ParB/Stage 0 sporulation protein J
fig|6666666.8381.peg.9	4506	5060	555	hypothetical protein
fig|6666666.8381.peg.10	5092	5304	213	hypothetical protein
fig|6666666.8381.peg.11	5421	5678	258	hypothetical protein
fig|6666666.8381.peg.12	5919	6155	237	hypothetical protein
fig|6666666.8381.peg.13	6174	6539	366	hypothetical protein
fig|6666666.8381.peg.14	6557	6793	237	hypothetical protein
fig|6666666.8381.peg.15	6945	7178	234	hypothetical protein
fig|6666666.8381.peg.16	7180	7575	396	hypothetical protein
fig|6666666.8381.peg.17	7637	8395	759	zinc metalloproteinase Mpr protein
fig|6666666.8381.peg.18	10832	8625	2208	hypothetical protein
fig|6666666.8381.peg.19	11125	11388	264	hypothetical protein
fig|6666666.8381.peg.20	11389	11631	243	hypothetical protein
fig|6666666.8381.peg.21	11639	13906	2268	VirB4
fig|6666666.8381.peg.22	13906	14925	1020	hypothetical protein
fig|6666666.8381.peg.23	14941	15093	153	hypothetical protein
fig|6666666.8381.peg.24	15086	15739	654	VirB8
fig|6666666.8381.peg.25	15736	16683	948	VirB9
fig|6666666.8381.peg.26	16685	17869	1185	trbI protein
fig|6666666.8381.peg.27	17894	19906	2013	hypothetical protein
fig|6666666.8381.peg.28	20033	20227	195	hypothetical protein
fig|6666666.8381.peg.29	20245	21192	948	ATPase required for both assembly of type IV secretion complex andsecretion of T-DNA complex, VirB11
fig|6666666.8381.peg.30	21176	21547	372	hypothetical protein
fig|6666666.8381.peg.31	21778	23223	1446	conjugal transfer protein (traG)
fig|6666666.8381.peg.32	23240	23449	210	hypothetical protein
fig|6666666.8381.peg.33	23450	23626	177	hypothetical protein
fig|6666666.8381.peg.34	23643	26366	2724	DNA primase traC (EC 2.7.7.-) (Replication primase)
fig|6666666.8381.peg.35	26347	27345	999	hypothetical protein

A putative protein involved in partitioning ParB/stage 0 sporulation was detected in ORF peg8 and showed 62% similarity to the transcriptional regulator involved in chromosome partitioning ParB in *A. butzleri* JV22. It also showed 62% homology to the plasmid replication-partition related protein in *H. pylori*. A putative zinc metalloproteinase (Mpr) was detected in ORF peg17, showing 58% similarity to the putative zinc metallopeptidase found in *Vibrio tubiashii* and in the conjugative tetracycline resistance plasmid pFBAOT6 detected in *Aeromonas punctata*. A similarity of 52% to the putative zinc metallopeptidase was also detected in different plasmids such as IncN R46 (Escherichia coli) and *Klebsiella pneumonia* plasmids. More ORFs were found in the *A. butzleri* plasmid, but no function could be attributed to those putative proteins (Table 3).

The genomic diversity of bacteria is caused by continuous genomic changes, such as horizontal gene transfer within and between bacterial populations, and intragenomic changes, such as rearrangements, insertions, point mutations, deletions, duplications and inversions. DNA insertions in *Arcobacter* have previously been reported in the 23S rRNA gene of *A. cryaerophilus* [21]. Plasmids are also one of the factors with a role in gene transfer. In this study, four small and one large plasmid were sequenced and annotated. The small plasmids carry replication proteins, which are necessary for replication and transfer of the plasmid in a new generation. A putative mobilization protein was also detected in those small plasmids showing a 46% similarity of to the mobilization protein in *Flavobacterium branchiophilum* and 42% similarity to that in *Campylobacter lari*. The features of mobilizable small plasmids could be of great importance in the development of recombinant *Arcobacter* strains. Investigation of the plasmid ability to exhibit horizontal transfer should be highlighted in the context of the development of modified strains.

Diguanylate cyclases (DGCs) are enzymes of second messenger signaling in bacteria. Their activity is responsible for the synthesis of the signaling compound cyclic di-GMP from two GTP molecules [27]. The abundance and importance of this gene in *Arcobacter* should be investigated. However, the catalytic and regulatory mechanisms of this class of enzymes are poorly understood. Cyclic di-3′,5′-guanylate is an intracellular signaling molecule that controls motility and virulence in bacterial cells. In Gram-negative bacteria production of cyclic di-3′,5′-guanylate (c-di-GMP) plays a role in the production of extracellular polysaccharides and biofilm formation [28], [29]. Furthermore the complete genome of *A. butzleri* shows also a cyclic-di-GMP factor [30]. Therefore, the diguanylate cyclase gene may be an interesting target for biofilm activity investigation.

The complete sequence of the large plasmid revealed a large number of putative genes similar to those involved in the mechanism of the type IV secretion system found in *W. succinogenes*. This type of secretion could play a role in DNA transfer and also protein or toxin secretion. Seven genes on the large plasmid that encode putative type IV secretion proteins are clustered in a region spanning 11.6 kbp with an overall G+C content of 27.3%. The Orthologs of genes VirB4, VirB6, VirB8, VirB9, VirB10, VirB11 and conjugal transfer protein TraG Like are located respectively in ORFs peg21-22-24-25-26-29 and peg31. VirB4 and VirB11 both contain nucleotide binding domains and exhibit ATPase activity. VirB8, VirB9, and VirB10 are putative pore-forming proteins components of type IV secretion system [14], [31], [32]. A similar TraG-like protein was also detected in this plasmid (ORF peg31). This gene was associated with the type IV secretion and also participates in DNA transfer [33] of *C. jejuni* invasion into epithelial cells. Furthermore this TraG-like associated with translocation cytotoxin CagA protein in *H. pylori* [34] and also shows similarity to TraG located in pTet plasmid in *C. jejuni*, which was dispensable for invasion into epithelial cells [35], [36]. The putative TraG-like protein also shows homology to the coupling protein VirD4 found in *W. succinogenes* and *C. jejuni* plasmid pVir (Figure 2). The presence of the putative genes VirB4, VirB9, VirB10, VirB11, and VirD4 suggest the presence of a potential functional type IV secretion machinery in this plasmid (Figure 2).

Plasmids are capable of autonomous replication. The annotation of the large plasmid shows a putative gene located in ORF 34 showing a homology with replication primase in *Y. pseudotuberculosis* and *H. pullorum* suggesting that this protein is responsible for replication of this plasmid.

*Arcobacter* is a very heterogeneous genus, and especially the species *A. cryaerophilus is* known for its large strain diversity. The failure to determine the sources of contamination and the huge genotypic diversity of arcobacters has been previously reported [22], [37]–[40]. The presence of several mobilizable or conjugatives plasmids may play a role in the genetic variability and diversity of *Arcobacter.* The putative type IV secretion system could play an important role in gene transfer within *Arcobacter*.

In conclusion, the four small plasmids are good candidates as modified vector to investigate for phenotypic and genotypic analysis to identify their role in arcobacters. The large plasmid could be used for genetic investigation and gene transfer, especially the investigation of the type IV secretion system in the exchange of DNA and protein and also their automobilization. The putative self-mobilizable plasmid (AC1119) could be a potential plasmid to investigate the virulence factors of this strain using *in vitro* models.
